# Role of the Scaffold Protein MIM in the Actin-Dependent Regulation of Epithelial Sodium Channels (ENaC)

**Published:** 2018

**Authors:** L. S. Shuyskiy, V. V. Levchenko, Y. A. Negulyaev, A. V. Staruschenko, D. V. Ilatovskaya

**Affiliations:** Institute of Cytology of RAS, Tikhoretskij Ave. 4, St. Petersburg, 194064, Russia; Department of Physiology, Medical College of Wisconsin, 8701 Watertown Plank Road, Milwaukee, WI 53226, USA; Department of Medical Physics, Peter the Great St. Petersburg Polytechnic University, Politekhnicheskaya Str. 2, St. Petersburg, 195251, Russia; Medical University of South Carolina, Department of Medicine, Division of Nephrology, 96 Jonathan Lucas St, MSC 629 CSB 822, Charleston, SC 29425, USA

**Keywords:** ENaC, MIM, cortactin, Arp2/3 complex, cytoskeleton

## Abstract

Epithelial Sodium Channels (ENaCs) are expressed in different organs and tissues, particularly in the cortical collecting duct (CCD) in the kidney, where they fine tune sodium reabsorption. Dynamic rearrangements of the cytoskeleton are one of the common mechanisms of ENaC activity regulation. In our previous studies, we showed that the actin-binding proteins cortactin and Arp2/3 complex are involved in the cytoskeleton-dependent regulation of ENaC and that their cooperative work decreases a channel’s probability of remaining open; however, the specific mechanism of interaction between actin-binding proteins and ENaC is unclear. In this study, we propose a new component for the protein machinery involved in the regulation of ENaC, the missing-in-metastasis (MIM) protein. The MIM protein contains an IMD domain (for interaction with PIP_2_ -rich plasma membrane regions and Rac GTPases; this domain also possesses F-actin bundling activity), a PRD domain (for interaction with cortactin), and a WH2 domain (interaction with G-actin). The patch-clamp electrophysiological technique in whole-cell configuration was used to test the involvement of MIM in the actin-dependent regulation of ENaC. Co-transfection of ENaC subunits with the wild-type MIM protein (or its mutant forms) caused a significant reduction in ENaC-mediated integral ion currents. The analysis of the F-actin structure after the transfection of MIM plasmids showed the important role played by the domains PRD and WH2 of the MIM protein in cytoskeletal rearrangements. These results suggest that the MIM protein may be a part of the complex of actin-binding proteins which is responsible for the actin-dependent regulation of ENaC in the CCD.

## INTRODUCTION

In epithelial cells, microfilaments (MF, fibrillar actin, or F-actin) are involved in the regulation of cell contacts, the formation of lamellipodia and filopodia, modulation of ion channel activity, and other processes [1, 2]. The cytoskeleton is directly or indirectly (with involvement of actin-binding proteins) associated with the cytoplasmic regions of ion channels and regulates their gating properties, incorporation, internalization, etc. [3-11]. Direct interaction between the cytoskeleton and epithelial sodium channels (ENaC) [11-14], aquaporin-2 (AQP2) water channels [15-17], CFTR channels [18-20], etc. has been shown. Cytoskeletal reorganization has an impact on the activity of ion channels [7, 21-24]. The effect of cytochalasin D leads to an increase in the ENaC open-state probability (*P_o_*) [10]. It is assumed that it is rather short microfilaments – but not globular actin (G-actin)or the long fibrils of F-actin – that regulate the activity of various ion channels [5, 10, 25, 26].

ENaCs belong to the DEG/ENaC (degenerin/epithelial sodium channels) superfamily. These channels are expressed in various organs and tissues in humans and animals (epithelium of the kidneys, lungs, intestines, etc.) and are responsible for sodium ions transport into the cell. A distinctive feature of DEG/ENaC channels is that they are inhibited by a nanomolar concentration of a diuretic amiloride [27]. According to current concepts, functional ENaC channels consist of three subunits: α, β, and γ, the ratio being 1 : 1 : 1 [28, 29]. In the kidneys, ENaC is expressed in the epithelial cells of the CCD, where it mediates the reabsorption of sodium ions and plays an important role in maintaining salt-water homeostasis and regulating blood pressure [30, 31]. ENaCs were found to co-localize with actin filaments [14, 32] and actin-binding proteins (ankyrin, spectrin, etc. [33]). Interaction of the channel with the α-spectrin SH3 domain via the proline-rich site at the C-terminus of the ENaC α-subunit has been shown [25, 33, 34]. The existing model of ENaC regulation is being constantly supplemented with new data: it was recently established that the cytoskeleton-binding protein ankyrin-G participates in the delivery of ENaC to the cell’s apical membrane in CCD [35]. We have proposed a model where cortactin (with involvement of the Arp2/3 complex) is the link between the channel and the cytoskeleton of CCD cells in mouse kidneys [36]. The interaction between ENaC and the cytoskeleton through adaptor proteins plays an important functional role in the regulation of the reabsorption of sodium in distal nephron.

**Fig. 1:**
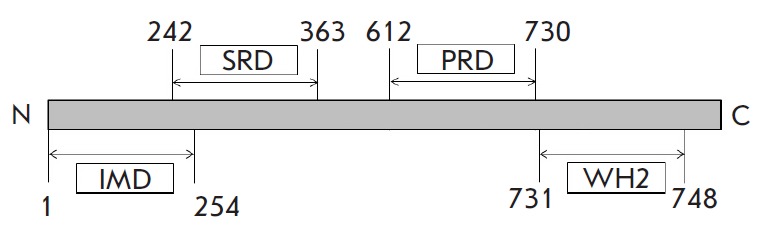
Domain structure of the mouse MIM protein (encoded by *mtss1*, UniProt Q8R1S4). The IMD domain can bind to F-actin, PIP2 rich membrane areas and Rac GTPases, and also plays an essential role in the dimerization of MIM. The SRD domain contains sites of Tyr phosphorylation. The PRD domain interacts with cortactin and tyrosine phosphatase delta. The WH2 domain binds G-actin

The adapter protein MIM (missing-in-metastasis), which is encoded by the *mtss1 *(metastasis suppressor 1) gene, was discovered in 2002. MIM, which was originally thought to be an actin-binding protein [37], is a significant element in the metastasis of several types of malignant neoplasms. MIM has been determined as a transcript absent in metastatic SKBR3 breast cancer cells and metastatic prostatic adenocarcinoma cell lines (LNCaP and PC3) [37-39]. MIM was assumed to function as a suppressor of metastasis [37]. However, there is still no definitive opinion on this point [40, 41]. An increase in MIM expression levels has been found to correlate with certain types of malignant transformations: for instance, in melanoma and head and neck squamous cell carcinoma [42, 43]. An increase in MIM expression also correlates with hepatocarcinoma progression [44]. MIM includes several important domains, which appear to play a key role in interactions with other proteins (see *Fig. 1*). For instance, the N-terminal domain of IMD (IRSp53-MIM homology domain) binds actin filaments, PIP2-rich membrane regions, small Rac GTPases and participates in protein dimerization. The SRD domain (serine-rich domain) contains tyrosine phosphorylation sites; the PRD domain (proline-rich domain) binds to cortactin and tyrosine phosphatase delta; the C-terminal domain WH2 (WASP homology domain 2) binds G-actin. MIM is presumably involved in cytoskeleton regulation through two independent actin-binding domains: IMD and WH2 [37, 39]. Co-localization of MIM with cortactin has been shown, as well as their apparent interaction with the proline-rich domain (PRD) of MIM [45]. MIM is involved in cytoskeleton rearrangements [38, 45, 46]: increased expression of MIM is accompanied by the formation of actin-rich protrusions resembling ruffles and microspikes [47]. In mouse kidney epithelial cells, MIM is co-localized with the Arp2/3 complex, where it can mediate the assembly of actin filaments [48, 49]. Apparently, the functionally active protein is assembled into homodimers, with the IMD domain playing an important role in this process [50]. MIM is expressed in the kidneys of mouse embryos in the region of branching-collecting ducts, tubules, and glomeruli [51]. A significant expression level of MIM has been found in the cortical layer of newborn mouse kidneys, while a low MIM expression level has been shown in the brain area. Mice with a knockout *mtss1 *gene (MIM-/-) were born healthy: however, about half of the animals developed large and numerous cysts in their kidneys by the age of 5, with signs of an autosomal-dominant polycystic kidney disease in [51]. MIM modulates the interaction between the cytoskeleton and the plasma membrane and facilitates maintenance of cellular contacts in renal epithelium [52]. Taking into account the important role of the MIM protein in the functioning of renal epithelial cells, a question arises regarding the involvement of this protein in the regulation of ENaC activity. The aim of our work was to study the involvement of the MIM protein in the actin-dependent regulation of ENaC and extend the model to ENaC regulation by actin-binding proteins.

## EXPERIMENTAL

**Cell lines**

CHO (Chinese Hamster Ovary cells) cells of an immortalized line derived from Chinese hamster ovary epithelial cells (CHO-K1, American collection of cell cultures) were used in the study. The cells were cultured in Petri dishes in a DMEM medium supplemented with 10% fetal bovine serum and 80 μg/ml of gentamicin.

**Transient transfection**

Plasmids encoding the α, β, and γ subunits of mENaC [36, 53] and various forms of the mouse MIM protein (provided by Dr. Lappalainen and Dr. Zhao [45, 49, 54]) were used in the study. MIM full is the full-length protein; MIM PH is a chimeric protein containing an inactivated IMD domain conjugated to the PH (pleckstrin homology) domain of phospholipase C delta 1 (PLCD1) with impaired dimerization ability; MIM ΔPRD is a protein lacking the PRD domain (Δ617–727), which does not interact with cortactin; MIM ΔWH2 is a protein lacking the WH2 domain (Δ746–759), which does not polymerize G-actin; MIM/IMD-L is a plasmid that only encodes the long splice variant of the IMD domain, which is incapable of interacting with Rac GTPases (the rest of the protein is absent). All of the MIM plasmids encode the mouse protein and are based on the pEGFP-N5 vector. All information on plasmid design is contained in previously published articles [49, 54]. Reorganization of the cytoskeleton was analyzed using transient transfection of cells with various plasmids encoding the MIM protein and its mutant forms, with GFP transfection serving as a control. For electrophysiological experiments, cells were passaged on 4 × 4 mm coverslips with a density reaching 50–60% confluency on the day of transfection. The cells were co-transfected with the α, β, and γ subunits of mENaC (1: 1: 1 ratio) and various forms of the MIM protein 24 h prior to the experiments. The weight ratio of plasmid DNA is as follows: α-mENaC, 0.33 μg; β-mENaC, 0.33 μg; γ-mENaC, 0.33 μg (total amount of mENaC-encoding plasmids, 1 μg); GFP in the control sample, 1 μg; MIM (with each of the forms carrying the GFP label), 1 μg. A total of 2 μg of plasmid DNA was used per transient transfection. All experiments were performed on CHO cells with the use of the PolyFect transfection reagent (Qiagen). GFP-encoding plasmid served as a marker of successful transfection in the control sample.

**Imaging of the cytoskeleton of fixed cells**

**Fig. 2:**
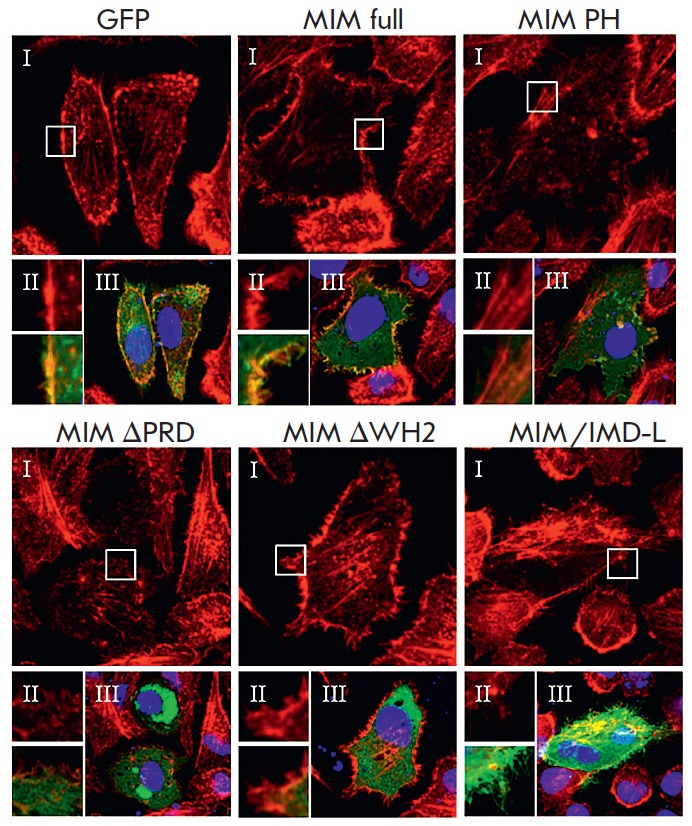
Actin cytoskeleton arrangement after transfection with different types of the MIM protein. Images of the actin cytoskeleton acquired with a confocal microscope (typical micrographs from 3 independent experiments) in fixed CHO cells after transient transfection with plasmids encoding different forms of the MIM protein (each plasmid based on pEGFP vector). *GFP *– control transfection; *MIM full *– full-length protein; *MIM PH *– chimeric protein, where the inactive IMD domain is conjugated with the PH domain of PLCD1, which leads to MIM’s inability to dimerize); *MIM *Δ*PRD – *the PRD domain (Δ617-727) of MIM is removed, and the protein cannot interact with cortactin; *MIM *Δ*WH2 *– the WH2 domain (Δ746-759) of MIM is removed, and this form of MIM cannot polymerize G-actin; *MIM/IMD-L – *an isolated long splice variant of the IMD domain (the rest of the MIM protein is absent), which cannot interact with Rac GTPases. I –rhodamine-phalloidine emission (red). II – magnified images of selected areas: upper panel – rhodamine-phalloidine emission, lower panel – merged image. III – merged image of GFP (green), rhodamine-phalloidine (red) and Hoechst-33342 (nuclear acids dye, blue) emissions

Fixation and staining of the transfected CHO cells was performed according to a standard protocol [36]. Cells were passaged on the coverslips (12 × 12 mm), washed with PBS the next day, and then fixed with 3.7% formaldehyde for 10 minutes at room temperature. Then, the cells were perforated with 0.1% Triton X-100 (5 min, room temperature) and incubated in a 2 μM rhodamine-phalloidin solution (Sigma-Aldrich) for 15 min at 37 °C. Nuclei were stained with a Hoechst-33342 dye (5 μg/ml, 5-min incubation, room temperature) and fixed on a slide using a Vectashield medium (Vector Laboratories). Addition of each reagent (pre-dissolved in PBS) was followed by washing with PBS. Imaging was carried out using a Nikon A-1R confocal microscope, ×100 lens, digital zoom. Lasers with excitation wavelengths of 405 nm (Hoechst-33342, emission maximum at 461 nm), 488 nm (GFP, emission maximum at 509 nm), and 561 nm (rhodamine-phalloidin, emission maximum at 565 nm) were used. Image analysis and processing were performed using the ImageJ software.

**Fig. 3:**
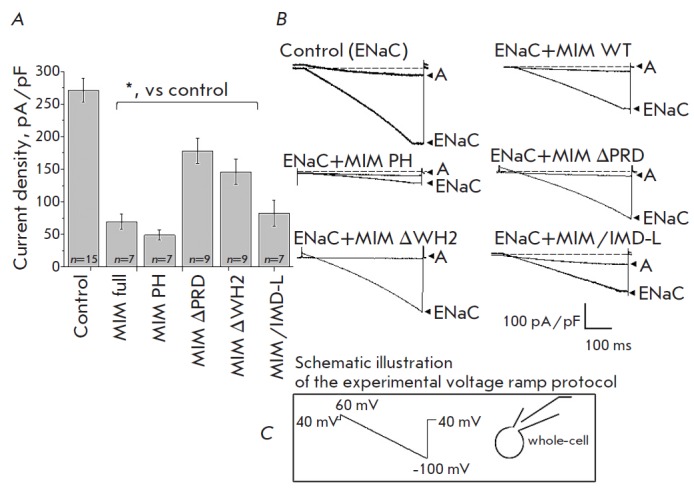
Effect of different forms of MIM on the amiloride-sensitive ENaC current density. *A *– summarized histogram of amiloride-sensitive current densities taken from electrophysiological experiments (patch-clamp in whole-cell configuration). CHO cells were co-transfected with mENaC plasmids, together with GFP (control), or mENaC with different types of the MIM protein (n – number of independent experiments; * – p < 0.05) *B *– representative traces of typical ENaC-mediated integral currents (ENaC – current magnitude, A – amiloride application at the end of the experiment (10 μM)). *C *– Schematic illustration of the experimental protocol

**Electrophysiology**

Integral currents were recorded using the patch-clamp technique in the whole-cell configuration. In order to determine the maximum value of the ENaC-mediated integral current, the experiments were performed under fluid shear-stress conditions; for the determination of the minimum value at the end of the experiment, the ENaC-mediated integral current was inhibited by the addition of amiloride (10 μM). An Axopatch 200B amplifier (Molecular Devices, Sunnyvale, CA, USA) connected via a Digidata 1440A A/D converter to a computer with installed pClamp 10.2 software (Molecular Devices) was used in the study. A Bessel filter (1 kHz) was used during the experiments. The currents were recorded at a fixed voltage using the previously described protocol [36] (schematic illustration of the voltage potential supply is shown in *Fig. 3C*): the potential was first held at +40 mV, followed by linear change from +60 mV to -100 mV (ramp, 500 ms duration). ENaC activity was defined as the current density value (current normalized to the cell capacitance) at -80 mV. Cells with a capacitance value in the range of 6÷10 pF were used for the analysis (the electrical capacitance of the cells was compensated prior to the experiment). Co-transfection with α-, β-, and γ-ENaC and a GFP-encoding plasmid (based on the pEGFP vector) was used as a negative control. The weight ratio of plasmid DNA was as follows: 1 μg of α-, β-, and γ-mENaC; 1 μg of GFP. Intracellular solution composition was as follows (mM): 120 CsCl, 5 NaCl, 5 EGTA, 2 MgCl_2_, 2 Mg-ATP, 40 HEPES/Tris; pH 7.4. Extracellular solution composition was as follows (mM): 140 LiCl, 2 MgCl_2_, 10 HEPES/ Tris, pH 7.4.

**Statistical analysis**

All results are presented as a mean ± standard error of the mean. Unpaired Student test calculated using the Microcal Origin 6.1 software (Microcal Software) was used for the analysis. Differences with *p* < 0.05 were considered statistically significant.

## RESULTS AND DISCUSSION

**The effect of various mutant forms of the MIM protein on the structure of the cytoskeleton**

We have studied the effect of the MIM protein (the domain structure of the protein is presented in *Fig. 1*) on cytoskeletal organization and ENaC activity. The effect of the MIM protein and its mutants on the cytoskeleton was analyzed in fixed CHO cells stained with rhodamine-phalloidin. The structure of the cytoskeleton in cells transfected with full-length MIM protein (*Fig. 2*, *MIM full*) was altered compared to the control transfection with GFP (*Fig. 2*, *GFP*): thickened actin filaments and formation of protrusions of the cell membrane (microspikes) in the sub-membrane region were observed. Transfection with the chimeric protein (*Fig. 2*, *MIM PH*) resulted in similar changes in the cytoskeleton structure, whereas transfection with the protein lacking the proline-rich domain (which does not interact with cortactin; *Fig. 2*, *MIM ΔPRD*) or the protein lacking the WH2 domain (which is not capable of polymerizing G-actin; *Fig. 2*, *MIM ΔWH2*) did not cause such changes. Transfection with the long splice variant of the IMD domain only (which is incapable of interacting with Rac GTPases; *Fig. 2*, *MIM/IMD-L*) led to an uneven distribution of the cytoskeleton compared to transfection with a full-length protein. Our results are consistent with the data obtained using 3T3 fibroblast cells [38], where transfection with MIM-GFP resulted in the appearance of abnormal worm-like actin structures and a reduction in stress fibers. Similar rearrangements of the cytoskeleton were observed after transfection with MIM/IMD-L (long variant of the IMD domain only) in a study of the IMD domain in U2OS cells [49]. A suggestion has been made that this results from the deformation of the plasma membrane. Thus, the cytoskeleton reorganizations identified in our study are associated with the PRD and WH2 domains of the MIM protein. Since MIM interacts with cortactin via the proline-rich domain (PRD), it can be assumed that MIM modulates cortactin-dependent and Arp2/3- mediated actin polymerization [52], which is important for various cellular functions, including the formation of cellular protuberances [49].

**Effect of the MIM protein on the ENaC-mediated integral current**

Dynamic rearrangements in the cytoskeleton are one of the mechanisms of ENaC activity regulation [14, 32]. According to data obtained by us using mouse kidney epithelial cells, the acting-binding proteins cortactin and Arp2/3 complex are involved in ENaC regulation [36]. MIM protein expression was detected in the kidney region expressing ENaC; its co-localization with cortactin and the proteins that form the Arp2/3 complex has been established [45, 52].

**Fig. 4:**
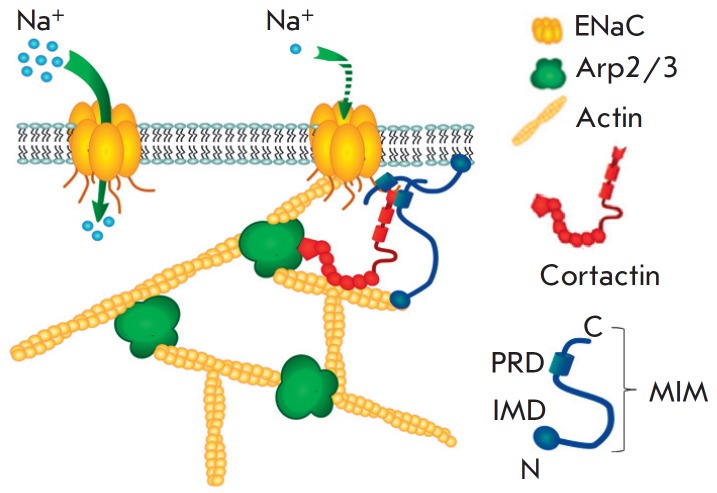
Suggested scheme of actin-dependent regulation of ENaC by the actin-binding proteins MIM, cortactin, and the Arp2/3 complex

The following density values of the integral ENaC-mediated current were obtained in electrophysiological experiments (pA/pF): control, 271.2 ± 18.3; co-transfection with MIM full, 69.6 ± 11.9; MIM PH, 48.9 ± 7.8; MIM ΔPRD, 178.0 ± 19.3; MIM ΔWH2, 146.0 ± 19.4; MIM/IMD-L, 82.7 ± 19.8. The summary diagram and representative current recordings are shown in *Fig. 3 A,B*. As seen in *Fig. 3A*, the ENaC-mediated current was significantly lower upon co-transfection of channel subunits with full MIM protein. In addition, we showed that all of the tested mutants significantly reduce channel activity compared to the control values when channel subunits are expressed without MIM proteins. However, mutant forms of MIM (ΔPRD and ΔWH2) had the weakest effect on the integral current density. Thus, we can assume that the MIM protein (alongside the actin-binding proteins cortactin and Arp2/3 complex) is involved in the actin-mediated regulation of ENaC. Based on the obtained data, a hypothesis (*Fig. 4*) has been proposed according to which a multifunctional adapter protein MIM is involved in the cytoskeleton-mediated regulation of ENaC.

## CONCLUSION

Blood pressure in the body directly depends on the homeostasis of sodium ions (Na^+^). This process is regulated by kidneys through the re-absorption of Na^+^ and water via various ion channels and transporters, including the epithelial sodium channels (ENaC) in the aldosterone-sensitive distal nephron. The decrease in the ENaC open probability, as shown earlier [36], may be due to a cortactin-dependent and Arp2/3-mediated reorganization of the cytoskeleton. However, the exact mechanism of ENaC activity regulation by the cytoskeleton and adaptor proteins is not yet fully understood. The MIM adaptor protein can be a new actor in the multicomponent model of ENaC regulation. We established that MIM is involved in the cytoskeleton-mediated regulation of ENaC activity and showed the important role played by the PRD and WH2 domains using the patch-clamp electrophysiological technique. The resulting images of the cytoskeleton confirm the participation of the MIM protein in the processes of cytoskeleton organization. Thus, it is apparent that the activity of ENaC is regulated by cytoskeleton rearrangements with the participation of a multi-protein complex which, alongside with cortactin and the Arp2/3 complex, may also include MIM (Fig. 4). Studying the fine-tuning of this complex is important for understanding the molecular mechanisms that may underlie many pathophysiological conditions.

## Abbreviations

ENaC: epithelial sodium channel
mENaC: mouse epithelial sodium channel
MIM (missing-in-metastasis): adaptor protein
mtss1: gene encoding MIM protein
